# Influence of a Lighting Column in the Working Width of a W-Beam Barrier on TB51 Crash Test

**DOI:** 10.3390/ma15144926

**Published:** 2022-07-15

**Authors:** Radoslaw Wolny, Dawid Bruski, Marcin Budzyński, Lukasz Pachocki, Krzysztof Wilde

**Affiliations:** 1 Department of Mechanics of Materials and Structures, Faculty of Civil and Environmental Engineering, Gdansk University of Technology, 80-233 Gdansk, Poland; dawid.bruski@pg.edu.pl (D.B.); lukasz.pachocki@pg.edu.pl (L.P.); krzysztof.wilde@pg.edu.pl (K.W.); 2 Department of Highway and Transportation Engineering, Faculty of Civil and Environmental Engineering, Gdansk University of Technology, 80-233 Gdansk, Poland; marcin.budzynski@pg.edu.pl

**Keywords:** road safety, field and numerical tests, road barriers, passive safety

## Abstract

Road equipment, such as, e.g., road safety barriers and lighting columns, are subject to certification according to the EN1317 standard to be allowed for use on European roads. In engineering practice, due to the terrain conditions, there are cases where other road equipment is installed within the working width of road safety barriers. Such situations are not considered during the certification process. Hence, the aim of this study is to analyze the effect of a lighting column installed within the working width of the barrier on the results of the TB51 crash test. The full-scale crash test and numerical simulation of this event were conducted. In the full-scale crash test, as well as in the simulation, the lighting column prevented the barrier’s post from properly disconnecting from the guardrail, which resulted in the barrier failing to restrain and redirect the 13-t bus. The simulation was quantitatively compared to the experiment, where the correlation coefficient of ASI curves equaled 84%. The THIV curves differed significantly between the experiment and the simulation, which is explained within the paper. Next, simulations with and without the lighting column were compared. The ASI and THIV in the simulation without the column were 0.33 and 16.1 km/h, respectively. In the simulation with the column, the ASI and THIV were 0.44 and 17.7 km/h, respectively. The maximum roll angle of the vehicle in the simulation without the column was 2.01° and with the column was 5.96°. The main difference, however, was that the system without the lighting column within the working width of the barrier was capable of properly restraining and redirecting the vehicle. The specific mechanics underlying this behavior are described within the paper.

## 1. Introduction

Road safety is one of the key aspects of life of a modern society. With the continuous development of road networks and with the increasing number of vehicles, a strong emphasis should be placed on the improvement of road safety. In 2021 alone, 19,800 people died on roads in the EU [1]. In Poland alone, on average, nearly 3000 people are killed on the roads each year, about 20% of them as a result of a vehicle falling out of its lane [2]. Those numbers show how much remains to be done in improving road safety. The key to understanding the need and building tools to manage road infrastructure to reduce the consequences of lane departure vehicle accidents is to know the actual conditions on the roads [3] and to identify the hazards and their sources that result from inadequate design, construction, installation, and maintenance of roadway vehicle restraints [4,5,6,7]. Roadway barriers should be considered as obstacles, and their use should be treated as a necessity [8,9,10]. This applies especially to bridges and locations where it is not possible to use a safety zone [11,12,13]. One way to improve this is to install road safety equipment in particularly hazardous locations around roads, i.e., road safety barriers (RSBs). In the EU, road barriers must pass crash tests according to the EN 1317 standard [14] to be approved for use on common roads. During the crash test, the barrier must demonstrate essential proprieties, i.e., it must properly restrain a vehicle and prevent the vehicle from overriding or tearing the barrier [15,16,17]. Supporting the field tests are numerical simulations that greatly expand the range of tested road barrier parameters [18,19,20]. Numerical analyses could be used to address specific issues, such as, e.g., problems such as disabled drivers [21]. The consequences of overriding or tearing the barrier are usually catastrophic, especially if an accident happens on a bridge or in the case where a bus is involved, as a number of passengers could be on board. One of the conditions for an effective operation of a road barrier is to provide an obstacle-free zone behind the barrier, so that, in the case of a vehicular crash, the barrier has sufficient space for deflection [22,23,24]. The size of that zone is defined by the working width and vehicle intrusion indices, which are determined according to EN 1317 in a full-scale crash test. However, on roads, it is often observed that other safety devices are installed directly behind the barrier, which may affect the barrier response. There are various reasons for such solutions. One of them may be the local constraints associated with the topology of the surrounding terrain and/or existing road infrastructure. Despite there being many studies on vehicular impacts into RSBs [25,26,27] or impacts into supporting structures [28,29,30], there are almost no studies investigating the influence of the installation of supporting structure in the barrier’s working width on the crash outcome. One of a few studies on that subject was performed by La Torre et al. [31]. That study analyzed how the installation of a variable message sign (VMS) behind a barrier can affect the crash results. The performance of the barrier will fully comply with the EN 1317 standard [14] requirements only if the VMS is placed at a distance of at least 130 cm from the front of the barrier as compared to the 200 cm working width of the device analyzed. However, many more studies are still needed to properly address that issue.

**Figure 1:**
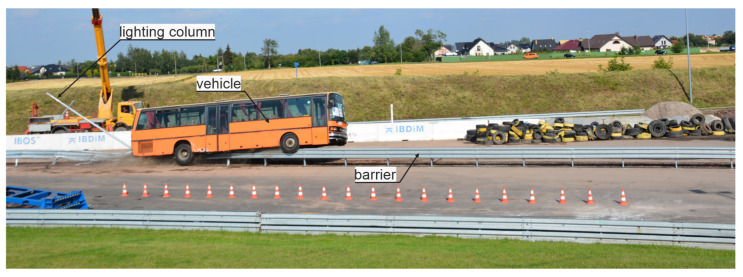
Exemplary view of the bus during the analyzed crash test.

This study analyzes the effect of the installation of a lighting column in the working width of a W-beam RSB on the results of the TB51 crash test, as in Figure 1. This research is important as it will shed light onto the mechanisms and interactions that occur in the barrier–column–vehicle system during an accident. This will allow the verification of whether two road safety devices that are safe separately will also work together to achieve an appropriate level of safety. The TB51 crash test was selected as it involves a 13-tonne bus, which can inflict serious damage to the barrier during the impact. Moreover, this particular impact can have extremely tragic consequences, as there may be many fatalities and seriously injured persons. The W-beam barrier was chosen as it is one of the most frequently used types of barriers on roads in the EU. That barrier successfully passed full-scale crash tests according to EN 1317 [14]; hence, it has appropriate certificates for use on European roads. Similarly, the lighting column was positively tested in accordance with the EN 12767 standard [32]. However, the tests for both systems were conducted separately. Hence, the research aimed to evaluate the safety of the combination of those two road safety systems: the W-beam barrier and the lighting column. The other objectives of this study were as follows:

- To develop a numerical model of the crash test and its validation for two cases: (1) the test with the barrier and the lighting column and (2) the test with the barrier alone;
- To analyze the results of the crash tests;
- To evaluate the influence of the lighting column on both: the barrier performance and the behavior of the bus.

## 2. Materials and Methods

In this study, a modified TB51 full-scale crash test was conducted. According to the EN 1317 standard, the TB51 test considers a 13-tonne bus that hits the barrier with a speed of 70 km/h at an angle of 20°. The modification of the test considered the installation of a lighting column within the working width of the system. The RSB was certified for use on European roads and obtained a W4 class of working width and a VI4 class of vehicle intrusion. The impact severity of the system was classified to the A class, and the system’s containment level was H2. The numerical model of the RSB was validated against a normative TB51 crash test, the results of which were obtained from the system’s manufacturer. Additionally, the model was also validated against the modified TB51 crash test, which is presented in the current work.

For the ease of description, the following nomenclature is introduced for the tests:

- Case No. 1—a simulation with a lighting column;
- Case No. 2—a simulation without a lighting column.

### 2.1. Full-Scale Experiment

**Figure 2:**
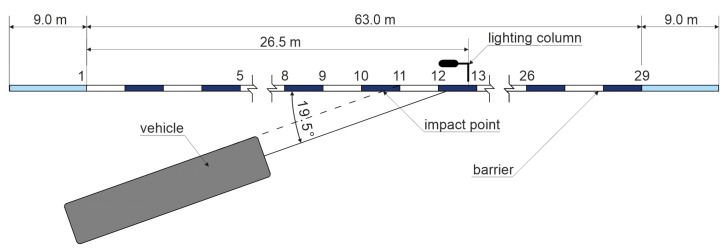
Scheme of the test setup.

The full-scale crash test was conducted on 10 August 2017 at the testing grounds of the Research Institute for Protective Systems (IBOS) [33]. The experiment was carried out by the Research Institute for Roads and Bridges (IBDiM) [34]. This institute is certified by the Polish Center for Accreditation to, among others, perform crash tests according to the EN1317 standard [14]. The setup of the test is presented in Figure 2. The total length of the system was 81 m, where there was 63 m of straight section and 9 m at each end of the barrier.

**Figure 3:**
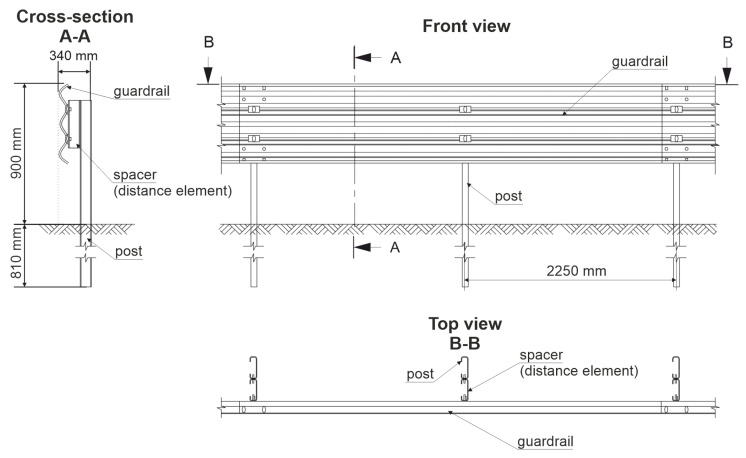
Technical drawing of the analyzed H2/W4/A road safety barrier.

A segment of the barrier is presented in Figure 3. The system consisted primarily of three main parts, that is a guardrail, posts, and spacers. The length of the guardrail section was 4.82 m. The total width of the barrier was 0.34 m. The height of the system equaled 0.90 m from the level of the ground. The posts were spaced every 2.25 m. Their total length was 1.71 m, where 0.81 m was anchored in the ground. The soil on the test site contained broken aggregate 0–31.5 mm. Its compaction factor was equal to 1.00, and the bearing factor was in the range of 65 to 90%.

**Figure 4:**
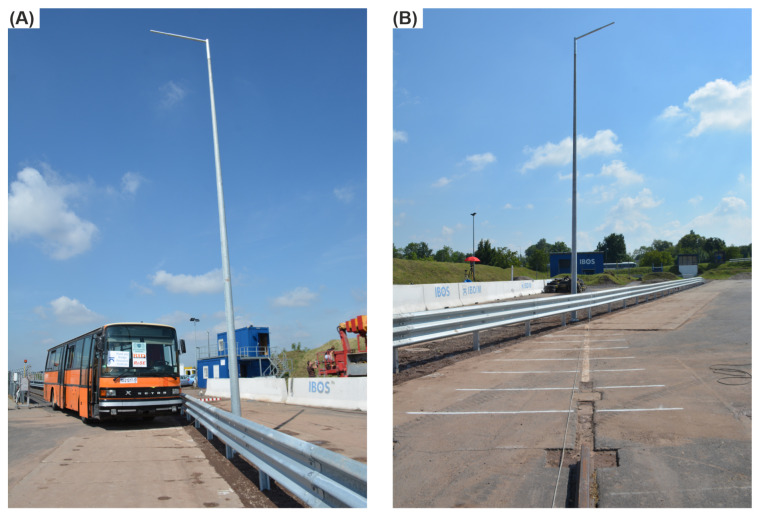
Test site before the collision, with (**A**) a view of the run-up track with the bus and the impact point and (**B**) a view of the impact point from the point of view of the run-up track.

The analyzed crash test also considered the lighting column in the working width of the barrier. This column was the 100HE3 class according to the EN 12767 standard [32]. Without a foundation, the column was 10 m long and weighted 87 kg. It was mounted 1.75 m behind the 12th post of the barrier (see Figure 4), and its axis was distanced at 0.60 m from the face of the system. The foundation was made of a 150 × 30 × 30 cm reinforced concrete cube, weighing approximately 225 kg.

### 2.2. Numerical Simulation

#### 2.2.1. Road Safety Barrier

The numerical model of the H2/W4/A RSB was created using the documentation from the full-scale crash test and the manufacturer. Guardrails, posts, and spacers were modeled using fully integrated shell elements of a size of approximately 15 mm. Those elements were made of steel, and they had a piecewise linear plasticity material model assigned with the properties of S235 structural steel. The material properties of the steel were obtained from tensile tests conducted on specimens cut from a section of the considered RSB. Bolt connections were modeled using solid elements with reduced integration. They had the material law dedicated to spot weld connections, which is a common approach in FE modeling [35,36]. The material characteristics of the bolts corresponded to the bolts class 8.8, according to the ISO 4032-8 standard. Steel parts of the system had a failure criterion that was based on the maximum effective plastic strain of an element. The critical value of the plastic strain was assigned to each part of the barrier based on parametric analyses.

**Figure 5:**
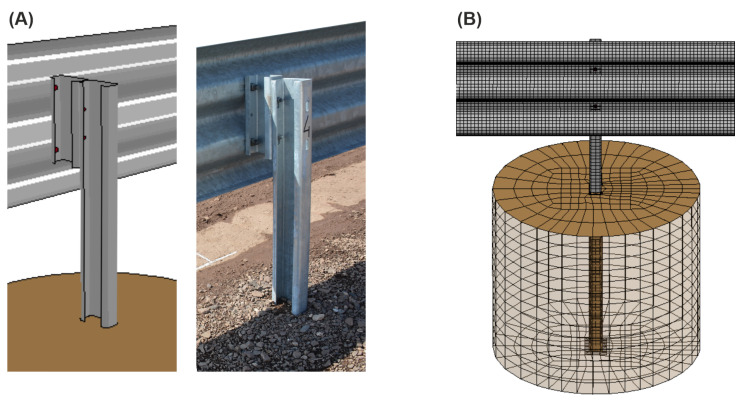
(**A**) Comparison between the actual post of the road safety barrier and the corresponding numerical model; (**B**) detail of FE discretization of a guardrail, a post, and the ground.

**Figure 6:**
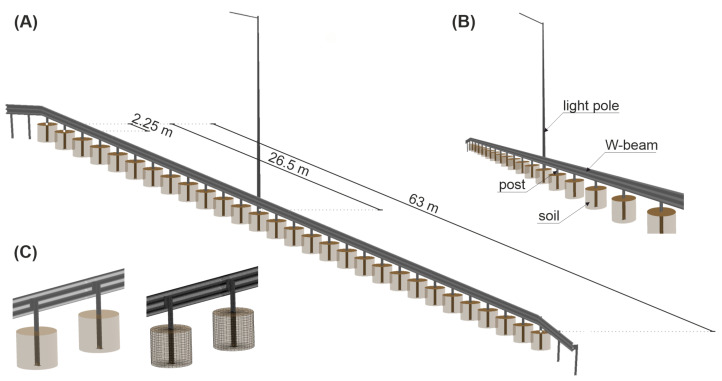
General views of the road safety barrier and the lighting pole with (**A**) specific dimensions, (**B**) a description of specific elements, and (**C**) a discretization detail of the barrier’s segment.

The model of RSB consisted of 271,018 nodes and 259,336 mainly quadrilateral FEs. The comparison between the selected part of the numerical model and the actual barrier is shown in Figure 5A. In Figure 5B, there is a view on the detail of the finite element discretization of the surroundings of the barrier’s post. Figure 6A,B show the numerical simulation setup and include descriptions of the objects involved. In Figure 6C, there is also a discretization detail of the barrier’s segment.

#### 2.2.2. Lighting Column

The lighting column model was created based on the column used in the experiment. The column in the test setup is shown in Figure 6A,B. The numerical model consisted of 3715 nodes comprising 3706 FEs. The elements of the column were modeled using four-node reduced integration shell elements. They had assigned a constitutive model of a piecewise linear plastic material law with material characteristics corresponding to S235 steel. The size of the elements was in the range from 20 to 50 mm, depending on the height. The mass and geometry were assigned according to the report from the experiment.

#### 2.2.3. Ground

In the simulation, the ground was modeled as cylinders surrounding individual posts; in which the posts were embedded. The shape of the post was cut from the soil cylinder so that the ground perfectly surrounded the barrier element. The interaction between the soil and the post was modeled using a penalty-based contact [37] with static friction of 0.4, dynamic friction of 0.2, and decay coefficient of 0.001, similar to [38]. Soil cylinders had a diameter of 1 m and a depth of 0.86 m. The cylinders of the ground were discretized using 8-node reduced integration solid elements. The view of the post and soil cylinder, with their corresponding discretization, is presented in Figure 5B and Figure 6C. The density of the ground was 2200 kg/m^3^; its bulk modulus was 97.75 MPa, and its shear modulus equaled 58.64 MPa. The constants of the ground’s plastic yield function constant were assumed as follows: $A_{0}=0.000811$, $A_{1}=0.0526$, $A_{2}=0.853$, and the pressure cut-off for tensile fracture was −0.3 MPa. These parameters were assumed based on previous work [35,36,39].

#### 2.2.4. Vehicle

**Figure 7:**
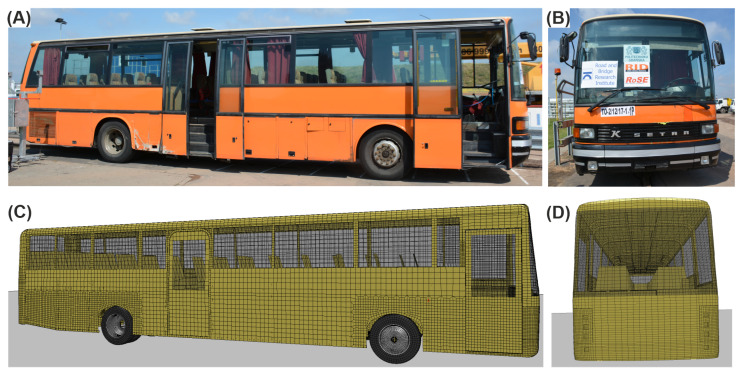
Comparison between the bus used in the experiment and the corresponding numerical model, with (**A**) a side view of the bus, (**B**) a front view of the bus, and (**C**) a side view and (**D**) a front view of the numerical model.

The bus model used in this study was developed by The Norwegian Public Road Administration [40]. The model of the bus was modeled mainly using fully integrated 4-node shell elements and selectively reduced solids. Most parts of the bus used the piecewise linear plasticity material law; the tires had the Mooney–Rivlin hyper-elastic constitutive relation assigned. Figure 7 shows the comparison between the bus used in the full-scale crash test and the bus from the simulations. In Figure 7A,B, there is a side view and a front view of the bus used in the experiment, respectively. The corresponding views for the bus from the simulations are in Figure 7C,D. A summary of the vehicle parameters, along with the requirements for the TB51 crash test vehicle, for both the simulation and the experiment, is presented in Table 1. As seen, all requirements were met for both vehicles.

### 2.3. Impact Severity Indices

The analysis was conducted according to the EN1317 standard where one of the methods of assessing the severity of a collision inflicted on the occupants of the vehicle is the Acceleration Severity Index (ASI) [14]. The ASI is a dimensionless metric based on acceleration components acquired from the vehicle’s center of gravity. The acceleration components are normalized to critical values according to the following formula:

(1) $$ASI\left(t\right)=\sqrt{{\left(\frac{{\bar{A}}_{x}}{{\hat{a}}_{x}}\right)}^{2}+{\left(\frac{{\bar{A}}_{y}}{{\hat{a}}_{y}}\right)}^{2}+{\left(\frac{{\bar{A}}_{z}}{{\hat{a}}_{z}}\right)}^{2}},$$

where ${\bar{A}}_{x}$, ${\bar{A}}_{y}$, ${\bar{A}}_{z}$ are the components of the acceleration along the axes *x*, *y*, and *z*, respectively. Before application to the formula, the components are filtered with a four-pole phase-less Butterworth low-pass digital filter with a cut-off frequency of 13 Hz. The variables ${\hat{a}}_{x}^{}=12\;G$, ${\hat{a}}_{y}^{}=9\;G$, and ${\hat{a}}_{z}^{}=10\;G$ are the limit values of acceleration components along *x*, *y*, and *z*, where G=9.81 $m/s^{2}$. The curve of the ASI represents the ASI as a function of time. However, the final value of the ASI is calculated as the maximum value of the ASI curve during a collision.

Another severity index is the Theoretical Head Impact Velocity (THIV), which denotes an impact speed of a theoretical head at the moment of contact with a theoretical vehicle cabin [14]. The indices ASI and THIV are usually calculated for tests of passenger vehicles. In this study, these indices were obtained for a bus to provide an additional quantitative measure for validation.

## 3. Validation of the Numerical Model

### 3.1. Full-Scale Experiment

**Figure 8:**
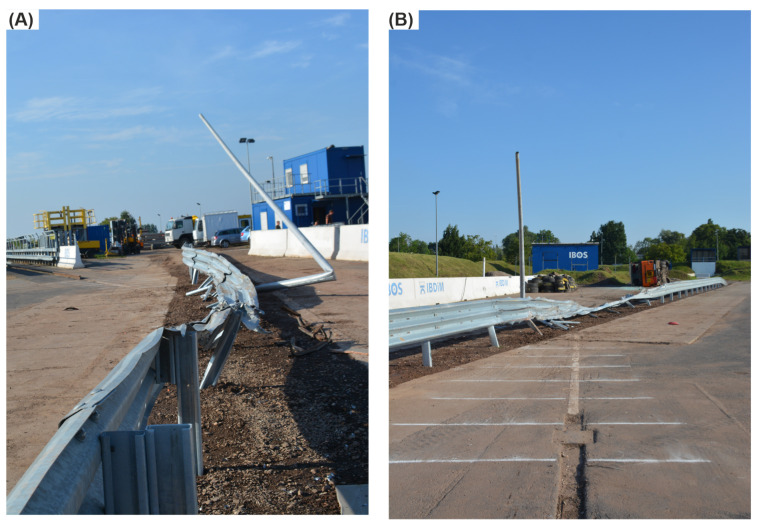
Test site after the collision with (**A**) a view from behind the barrier on the vehicle approach track and (**B**) a view from the approach track on the impact point.

The impact speed of the bus in the full-scale crash test was 73.2 km/h, and the angle equaled 19.5°, thus generating the vehicle’s kinetic energy of 298.7 kJ. During the experiment, the vehicle hit the barrier 10 cm before the post No. 11 and remained in contact with the guardrail. After passing the post No. 14, the bus ran over the barrier and rode along the barrier’s top up to the post No. 16. After reaching this post, the bus left the barrier on the side opposite to the in-run. At the height of the Post 29, the vehicle overturned to its left side, which can be seen in Figure 8B. In addition, damage to the protective system and the lighting column is presented in Figure 8A.

### 3.2. Numerical Simulation

The simulation was carried out using the MPP LS-Dyna R10.1 software with double precision on two 12-core Intel^®^ Xeon^®^ Processors E5 v3 @ 2.3 GHz (48 threads in total). The simulation of 2 s of the impact took 33 h and 46 min of computation time.

**Figure 9:**
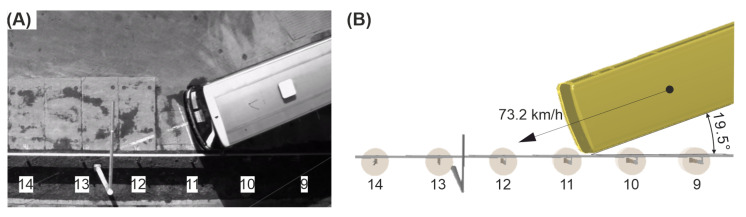
Impact conditions of the vehicle in (**A**) the full-scale crash test and in (**B**) the simulation.

The numerical simulation had the same impact conditions as in the experiment, i.e., the same velocity and the same impact angle. To impact location was also chosen as in the experiment; hence, the bus in the simulation hit the barrier 10 cm before Post 11. A comparison of the impact conditions between the experiment and the simulation is also presented in Figure 9.

**Figure 10:**
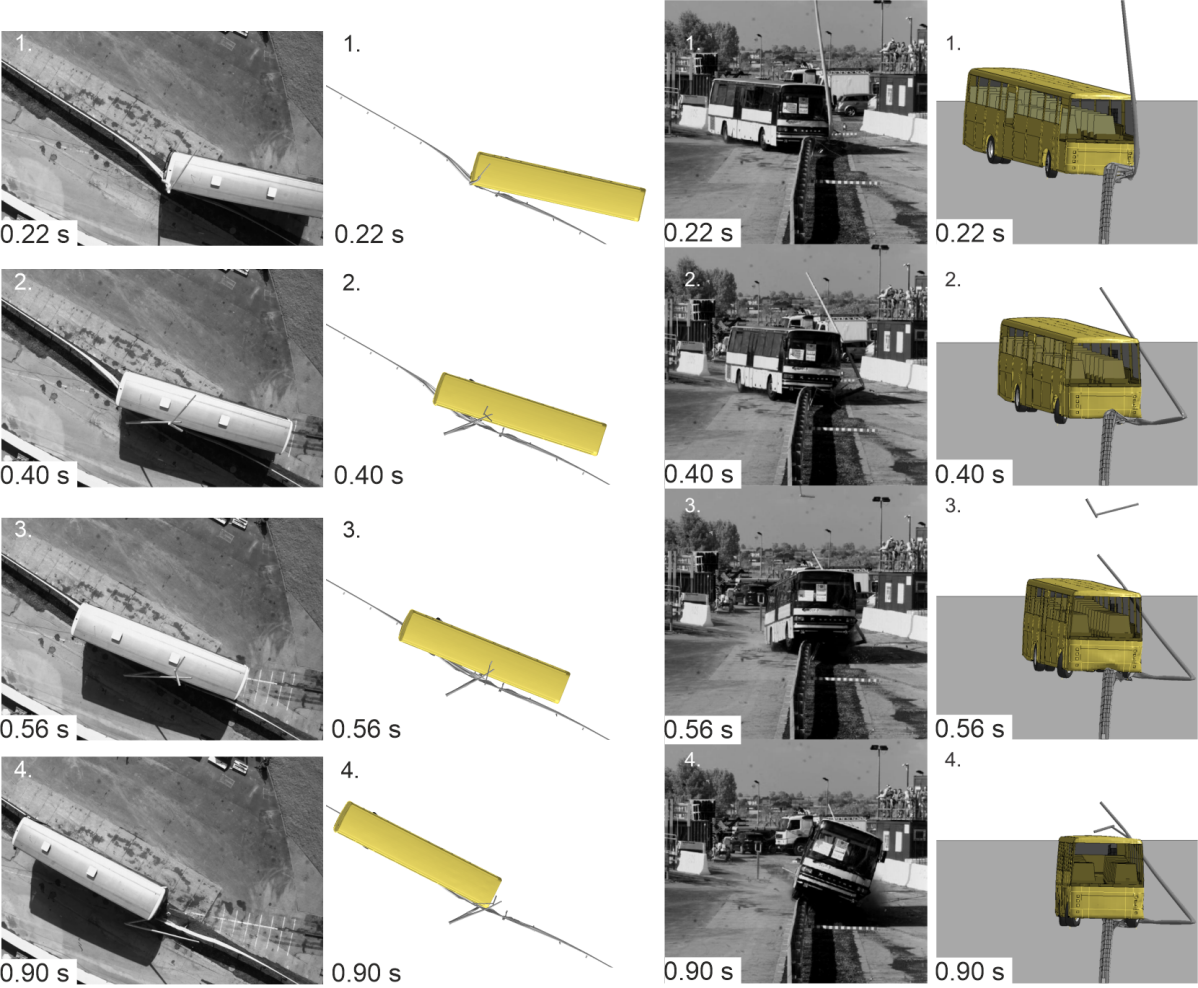
Vehicle trajectories of the full-scale crash test and the numerical simulation.

The results of the numerical simulation were compared with the corresponding experiment. A visual representation of the vehicle trajectories for both cases is presented in Figure 10. During the initial phase, the bus in the simulation and the experiment followed a similar path. Differences were observed in the latter parts of the test. The vehicle in the experiment hit the barrier and swung to its other side, as in Figure 10(2). As seen, the left side of the vehicle crossed the face of the barrier, and eventually, the whole bus followed and fully crossed over to the other side of the protective system (see Figure 10(4)). The wheels on the right side of the bus then caught the barrier, resulting in the bus rollover. A difference in the vehicle course was observed in the simulation where the center of the vehicle’s chassis did not cross the face of the barrier. Hence, as a consequence, the vehicle hung on the barrier, as seen in Figure 10(3). The simulation ended with the bus’s undercarriage hanging on the protective barrier, as in Figure 10(4). Nevertheless, in both, the experiment and the simulation, the analyzed road safety barrier did not stop the vehicle from crossing over the traffic lane.

**Figure 11:**
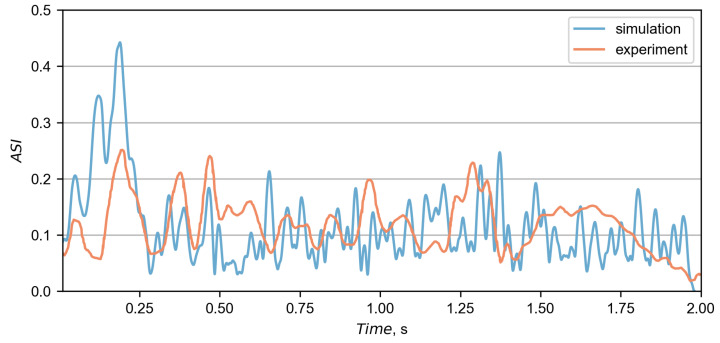
The comparison between the ASI calculated at the driver seat in the simulation and the experiment.

**Figure 12:**
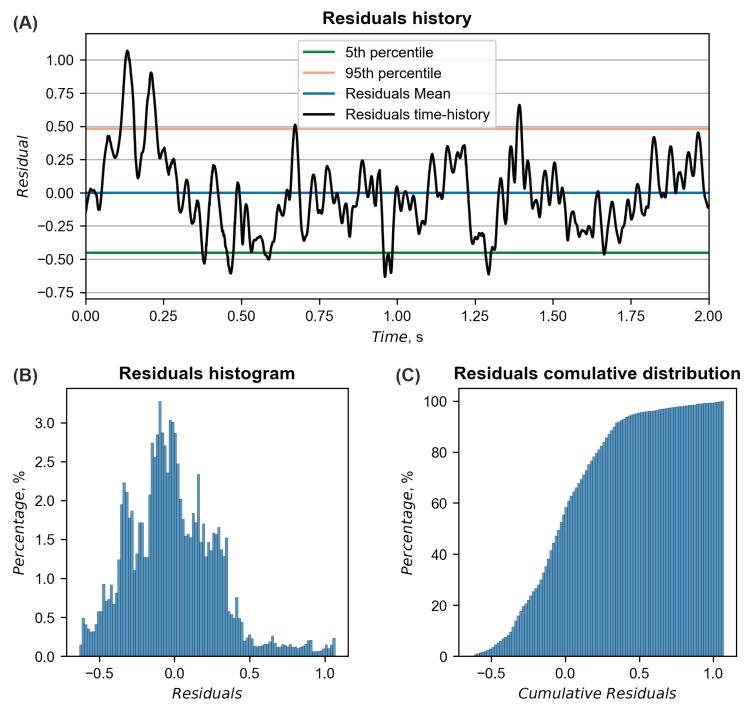
Residuals (**A**) history, (**B**) histogram, and (**C**) cumulative distribution of ASI curves.

The calculated ASI curves were based on accelerations from the driver seat and are presented in Figure 11. The similarity between the curves was calculated based on the MPC and ANOVA metrics, as proposed in the technical report [41]. Residuals of ASI curves with their corresponding distributions are presented in Figure 12. A summary of the metrics is in Table 2, where all criteria were fulfilled, indicating a good overall correlation between curves. The maximum ASI in the experiment was 0.25 and occurred 0.196 s after the initial impact. In the simulation, the maximum ASI was higher and exited 0.44 at 0.189 s.

**Figure 13:**
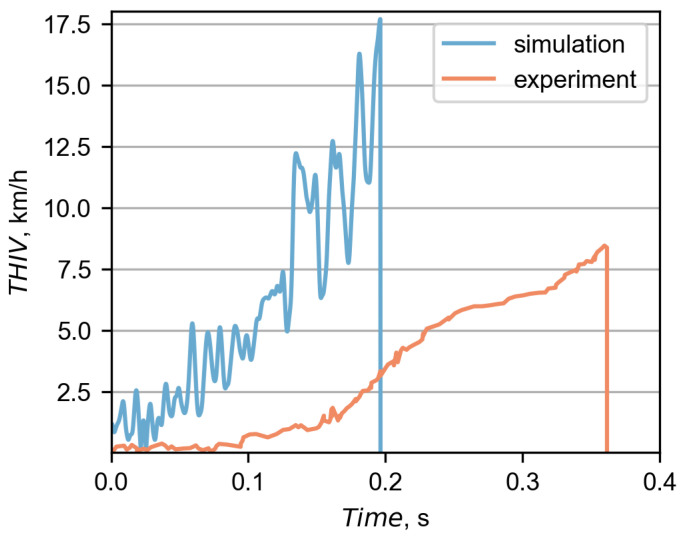
The comparison between the THIV calculated at the driver seat in the simulation and the experiment.

In Figure 13, there is a comparison between the experimental and the simulation THIV curves. For both cases, the THIV was based on accelerations from the driver seat. However, as seen in Figure 13, there are significant differences between those curves. Discrepancies are mainly due to the different rate at which the vehicle changed its direction after hitting the barrier. This is because the THIV index directly depends on the yaw velocity. In both cases, the barrier initiated the redirection of the bus. However, in the simulation, the redirection occurred faster and was more abrupt. In the experiment, the bus maintained its initial moving direction for longer. Additionally, the discrepancy may be due to the fact that in the simulation, the bus hanged on the barrier, and in the experiment, the bus completely ran over to the other side of the barrier.

To sum up this section, based on both the qualitative comparison of the crash test trajectories from the experiment and simulation and the quantitative comparison of specific metrics, the analyzed simulation was considered validated.

### 3.3. Damage of the Road Safety Barrier

As a result of the impact in the full-scale crash test, the system was damaged between Posts 11 and 19. For the simulation, the damage was between Posts 10 and 25. The guardrail was locally torn in the experiment at Posts 12, 15, 16, and 18. For the simulation, the guardrail was severely damaged at Posts 12, 15, 18, 19, and 20. In the full-scale crash test, Posts 11, 12, and 17 were bent, the posts between 13 to 16 were bent to the ground, and the posts from 1 to 11 were only twisted. For the simulation case, Posts 10, 11, 12, and 24 were bent, Posts 13 to 23 were bent to the ground, and the posts from 1 to 9 and No. 25 were only twisted. The lighting column for both the experiment and the simulation was damaged near the foundation and was broken at one third of its height.

**Figure 14:**
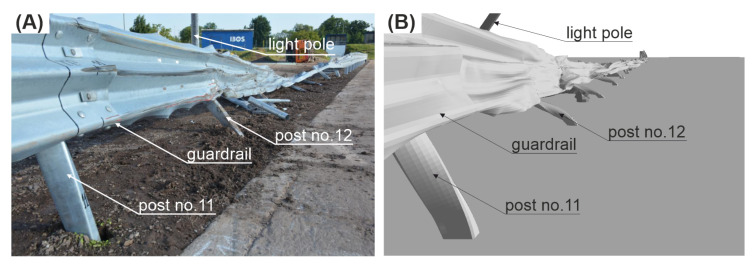
View of the damaged barrier. (**A**) Experiment; (**B**) simulation.

A view of the damage of the system can be seen in Figure 14.

### 3.4. Damage of the Vehicle

In the experiment, the rear window of the vehicle was broken, the front windshield was severely cracked, and the front bumper was crushed. Moreover, the left-front wheel of the vehicle was broken. The left side of the vehicle was damaged due to the contact with the guardrail. In the simulation, the left side of the vehicle was also damaged. Additionally, the front and rear bumpers were crushed due to the impact. Moreover, the cross-member of the chassis frame was damaged and the front axle was bent near the left-front wheel.

**Figure 15:**
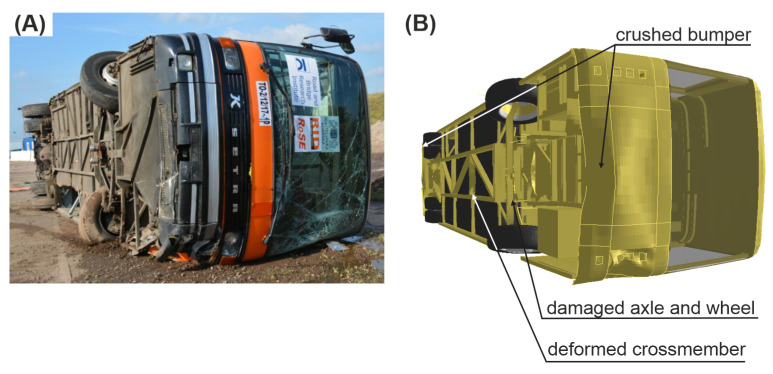
View of the damaged vehicle. (**A**) Experiment; (**B**) simulation.

A comparison of vehicle’s damage between the full-scale crash test and simulation is presented in Figure 15.

## 4. Collisions with and without the Lighting Column

**Figure 16:**
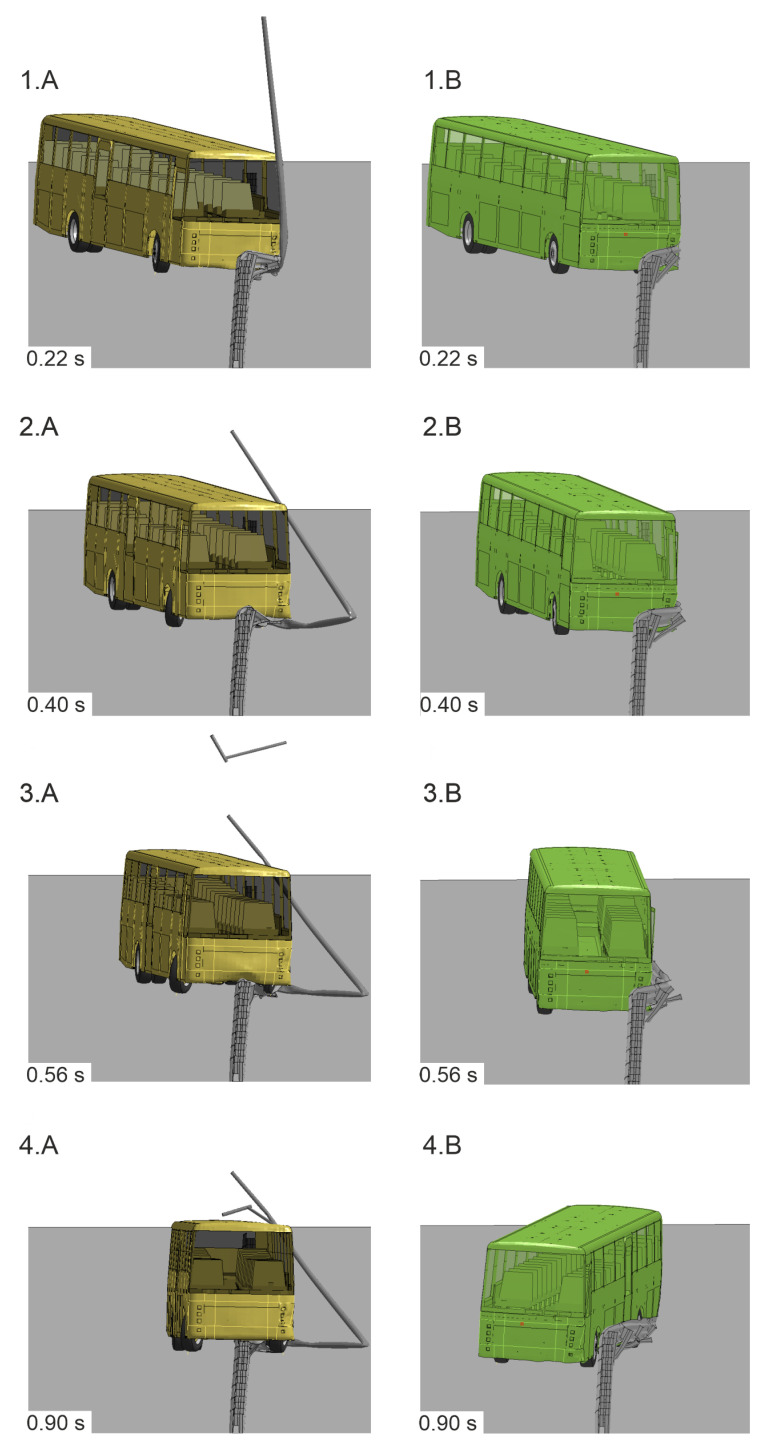
Vehicle trajectories of numerical simulations of (**A**) the barrier without an obstacle (Case No. 1) and (**B**) the barrier with a lighting column within its working width (Case No. 2).

Numerical simulations considered two cases, where Case No. 1 had the lighting column installed within the working width of the H2/W4/A W-beam RSB and Case No. 2 considered the barrier itself, without any obstacles. All of the modeling details were the same in both simulations, i.e., the model of the vehicle and the model of the barrier remained unchanged. Both cases had the same impact conditions, i.e., the impact speed of 70.6 km/h, the impact angle of 20.3°, and the impact location of 10 cm before Post No. 11. The course of the vehicles’ trajectories in both simulations is presented in Figure 16.

The trajectory of the vehicle in Case No. 1 was described in Section 3. In simulation Case No. 2, 0.08 s after the impact, the barrier guardrail flattened on the left-front side of the vehicle body and the vehicle began to slide and redirect along the guardrail. At 0.14 s after the impact, Spacer No. 12 was detached from the guardrail, and then, two more spacers (Nos. 13 and 14) also detached from the guardrail consequently. At 0.54 s after the impact, the rear of the bus hit the guardrail at the height of Post No. 10 (see Figure 16(3.B)). The rear of the vehicle was also caught by the barrier, and the guardrail properly redirected the vehicle; at 1.30 s after the impact, the vehicle eventually left the barrier. The calculated working width was 1.37 m, and the dynamic deflection was 1.03 m. The difference between the working width from the simulation and the value from its certificate was less than 10 cm. The maximum roll angle of the vehicle in the simulation Case No. 2 was 2.01°, and it was 3.95° less than in Case No. 1. A comparative analysis of the vehicle trajectories for both simulation cases is summarized in Table 3, where the time 0.0 s is the time of vehicle impact and the time 2.0 s indicates the end of the simulation.

**Figure 17:**
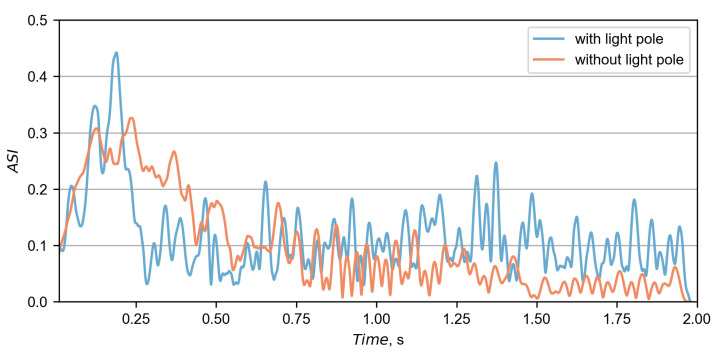
The comparison between the ASI calculated at the driver seat in both simulations.

**Figure 18:**
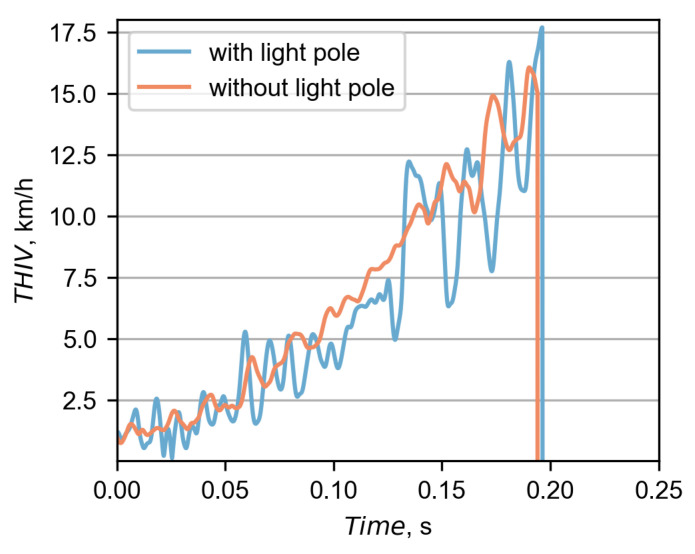
The comparison between the THIV calculated at the driver seat in both simulations.

The results of the ASI and THIV for both simulations were calculated based on accelerations from the driver seat. Their corresponding curves are presented in Figure 17 and Figure 18. In Case No. 2, the maximum ASI equaled 0.33 and was 25% lower than in Case No. 1. The time of the maximum ASI in Case No. 2 was at 0.235 s after the impact, which was 0.046 s later than in Case No. 1. The maximum THIV in Case No. 2 was 16.06 km/h at the time of 0.196 s, which is 9% less and 0.006 s earlier, compared to the other case.

**Figure 19:**
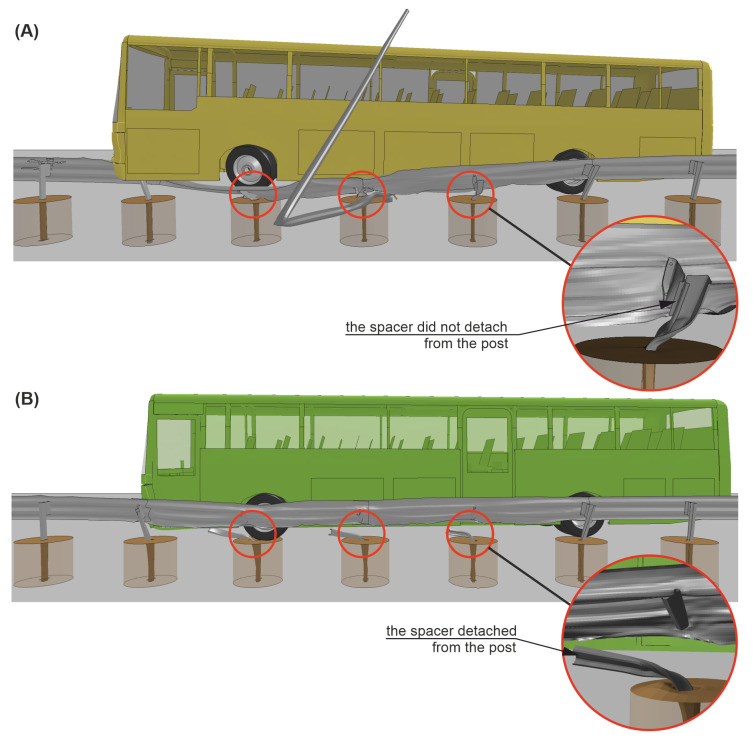
Side views of the H2/W4/B road safety barrier during simulations (time = 0.5 s) for (**A**) Case No. 1 and (**B**) Case No. 2. Note that the surface and soil cylinders are set transparent for clarity.

**Figure 20:**
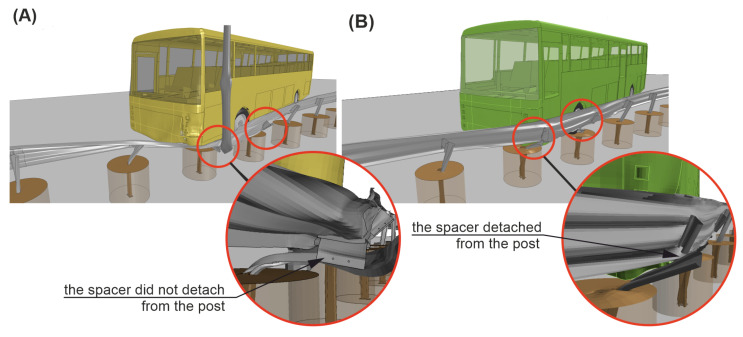
Isometric views of simulations (time = 0.3 s) for Cases (**A**) No. 1 and (**B**) No. 2.Note that the surface and soil cylinders are set transparent for clarity.

**Figure 21:**
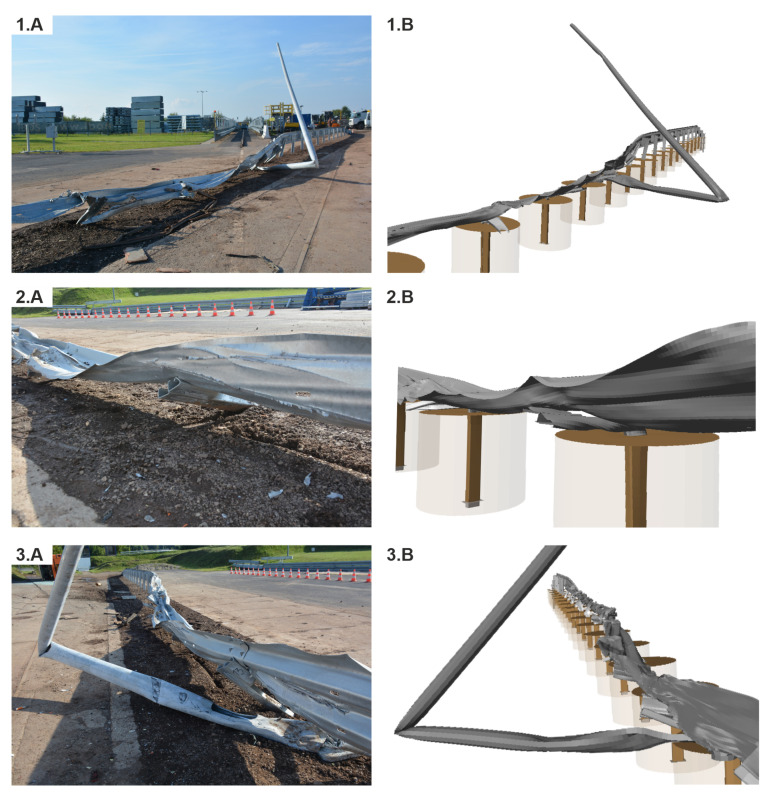
Views of the damaged H2/W4/A road safety barrier in (**A**) the experiment and in (**B**) the simulation. Note that the surface and soil cylinders are set transparent for clarity.

In Figure 19 and Figure 20, there is a comparison between Case No. 1 and Case No. 2 at crucial moments in the simulation. It was observed that Post Nos. 11, 12, and 13 in Case No. 1 (see Figure 19A) did not detach from the guardrail, in contrast to the ones in Case No. 2 (see Figure 19B). This difference seems to be crucial from the point of view of the correct operation of the system. The experiment additionally confirmed the simulation results, where also no detachment of the posts from the system was observed. The comparison of the damaged barrier in the experiment and corresponding simulation is presented in Figure 21.

## 5. Conclusions

This research showed that the influence of the installation of the lighting column in the working width of the H2/W4/A beam barrier led to the malfunction of the system during the TB51 crash test. In the considered case, the barrier did not properly restrain and redirect the vehicle.

It was found that the incorrect behavior of the system was due to the fact that the barrier posts did not properly disconnect from the guardrail at the appropriate moment. The failure of the post to disconnect prevented the barrier from working as a belt restraining the vehicle, and in this case, the post pulled the guardrail downwards. It was the lighting column that prevented the post from properly detaching itself from the guardrail, as the lighting column limited the unconstrained deflection of the system. Pulling the guardrail down caused it to form a ramp, which allowed the bus to run over to the other side of the system.

It is worth noting the fact that both analyzed systems, the barrier and the lighting column, had been previously certified for common use on European roads. Each of them individually is considered as safe road equipment; however, as presented in the current study, the combination of these systems may lead to dire consequences.

## Figures and Tables

**Table 1:** Comparison between the FE model of the bus, the bus used in experiment—SETRA S215 UL—and the requirements for the TB51 crash test, according to EN 1317.

	FE Model	SETRA S215 UL Model Year 1991	EN 1317
Mass	12,967.6 kg	12,992 ± 20 kg	13,000 ± 400 kg
Length	12.80 m	12.010 ± 0.020 m	n/a
Width	2.506 m	2.490 ± 0.020 m	n/a
Location of center of gravity (CG)	$CG_{x}$: 3987 mm $CG_{y}$: 0 mm $CG_{z}$: 1338 mm	$CG_{x}$: 3815 ± 2 mm $CG_{y}$: 3 ± 2 mm $CG_{z}$: 1435 ± 2 mm	$CG_{x}$: 3800 mm ± 10% $CG_{y}$: ±100 mm $CG_{z}$: 1400 mm +15%/−5%
Number of axles	2	2	n/a
Wheel base	6.798 m	6.080 ± 0.020 m	6.50 m ± 15%
Wheel track (front/rear)	2.088 m/1.769 m	2.070 ± 0.020 m/1.800 ± 0.020 m	2.00 m ± 15%
Wheel radius (front/rear)	0.495 m/0.495 m	0.490 ± 0.005 m/0.492 ± 0.005 m	0.52 m ± 15%
Number of nodes	125,550	n/a	n/a
Number of FEs	128,245	n/a	n/a

**Table 2:** The summary of MPC, ANOVA, and single value metrics.

MPC Metrics	Value, %
Sprague–Geers Magnitude	6.3
Sprague–Geers Phase	18.2
Sprague–Geers Comprehensive	19.2
**ANOVA Metrics**	
Average	−1
Std	29.6
**Single Value Metrics**	
Correlation Coefficient	84.1

**Table 3:** Description of the differences between the analyzed cases. (* the time of the impact, ** the end of the simulation.)

Time Intervals, s	Comparison between Simulations	
	Case No 1	Case No 2
0.0 *–0.13	**Similar Course**	
0.14–0.25	The guardrail made contact with the lighting column, which prevented the connection between the post and the guardrail from breaking (see Figure 16(1.A) and Figure 20A).	Post No. 12 was detached from the guardrail (see Figure 16(1.B) and Figure 20B).
0.26–0.39	Post No. 13 did not detach from the guardrail, and the system was pressed to the ground by the vehicle chassis (see Figure 16(2.A)).	Another post (No. 13) was detached from the guardrail. The guardrail flattened against the vehicle body and began to redirect the vehicle (see Figure 16(2.B)).
0.40–0.63	After sliding underneath the vehicle chassis, the guardrail was shaped into a flat surface parallel to the ground, which acted as a ramp, which allowed the vehicle to run over the barrier (see Figure 16(3.A)).	Post No. 14 disconnected from the guardrail, and the guardrail continued to effectively redirect the vehicle (see Figure 16(3.B)).
0.63–0.85	The left-front wheel of the vehicle crossed to the other side of the barrier, and the vehicle chassis landed on the top of the guardrail. The system underneath the bus almost completely laid down on the ground (see Figure 16(4.A) and Figure 19A).	The bus was moving parallel to the line of the barrier. Post No. 12, 13, and 14 were disconnected from the system (see Figure 16(4.B) and Figure 19B).
0.86–1.07	The left-rear wheel of the bus reached the barrier.	The vehicle continued to move along the guardrail.
1.08–2.0 **	The left-rear wheel of the bus crossed to the other side of the barrier, and the whole vehicle was over the system. The bus chassis pressed the system to the ground; however, no posts detached from the guardrail.	The bus eventually left the system completely redirected.

## Data Availability

The raw data supporting the conclusion of this article will be made available by the authors, without undue reservation.
